# Surgical Management of Massive Pericardial Effusion and Predictors for Development of Constrictive Pericarditis in a Resource Limited Setting

**DOI:** 10.1155/2016/8917954

**Published:** 2016-07-19

**Authors:** Emeka B. Kesieme, Peter O. Okokhere, Christopher Ojemiega Iruolagbe, Angela Odike, Clifford Owobu, Theophilus Akhigbe

**Affiliations:** ^1^Department of Surgery, Irrua Specialist Teaching Hospital, PMB 8, Irrua, Edo State, Nigeria; ^2^Department of Medicine, Irrua Specialist Teaching Hospital, PMB 8, Irrua, Edo State, Nigeria; ^3^Department of Paediatrics, Irrua Specialist Teaching Hospital, PMB 8, Irrua, Edo State, Nigeria; ^4^Department of Pathology, Irrua Specialist Teaching Hospital, PMB 8, Irrua, Edo State, Nigeria; ^5^Department of Radiology, Irrua Specialist Teaching Hospital, PMB 8, Irrua, Edo State, Nigeria

## Abstract

*Background*. The diagnosis and treatment of massive pericardial effusion and cardiac tamponade have evolved over the years with a tendency towards a more comprehensive diagnostic workup and less traumatic intervention.* Method*. We reviewed and analysed the data of 32 consecutive patients who underwent surgery on account of massive pericardial effusion and cardiac tamponade in a semiurban university hospital in Nigeria from February 2010 to February 2016.* Results*. The majority of patients (34.4%) were between 31 and 40 years. Fourteen patients (43.8%) presented with clinical and echocardiographic feature of cardiac tamponade. The majority of patients (59.4%) presented with haemorrhagic pericardial effusion and the average volume of fluid drained intraoperatively was 846 mL  ± 67 mL. Pericardium was thickened in 50% of cases. Subxiphoid pericardiostomy was performed under local anaesthesia in 28 cases. No postoperative recurrence was observed; however 5 patients developed features of constrictive pericarditis. The relationship between pericardial thickness and development of pericardial constriction was statistically significant (*p* = 0.004).* Conclusion*. Subxiphoid pericardiostomy is a very effective way of treating massive pericardial effusion. Removing tube after adequate drainage (50 mL/day) and treatment of primary pathology are key to preventing recurrence. There is also a need to follow up patients to detect pericardial constriction especially those with thickened pericardium.

## 1. Introduction

Massive pericardial effusion and cardiac tamponade are life-threatening cardiac pathologies that require urgent intervention.

The challenges in managing these conditions are not just only in treatment, but also in identifying the aetiological agent. In developing countries, the dominant cause of massive pericardial effusion is tuberculosis whereas in developed countries it is more likely to be caused by cancer, infectious, iatrogenic, connective tissue diseases and perhaps, in a good number of patients, the cause remains idiopathic [1, 2].

The nature of effusion drained from the pericardial space may be serous, haemorrhagic, or purulent. It may be massive, usually caused by malignancy followed by uraemia in the developed world [3]. A large effusion becomes a powerful predictor for development of cardiac tamponade when it has a circumferential echo space of more than 1 cm anteriorly and posteriorly [4].

Cardiac tamponade is a clinical emergency. Patients with cardiac tamponade present with features of elevated systemic venous pressure and the diagnosis is confirmed echocardiographically by right atrial or right ventricular diastolic collapse.

Patients that present with this condition should ideally be well investigated to diagnose the cause. Where facilities are available, pericardial fluid analysis for tumour markers (carcinoembryonic antigen, carbohydrate antigen CA-125, 19-9) is important in patients with suspected malignant effusion. In those with suspected tuberculous pericardial effusion, adenosine deaminase (ADA), interferon-gamma, PCR analysis for tuberculosis, and pericardial lysozymes should be done in addition to the routine pericardial fluid acid-fast bacilli staining and mycobacterium culture. High ADA level may predict the evolution towards constriction [5]. GeneXpert-MTB/RIF assay has been highly recommended as an initial diagnostic platform for early and quick detection of TB cases and hence can be very useful in diagnosing TB pericardial effusion [6]. Pericardioscopy can be useful in obtaining pericardial biopsy [5]. However in the setting of a developing country, there is significant challenge acquiring and performing the entire investigative armamentarium required for diagnosing the cause of massive pericardial effusion.

Pericardiocentesis which can be percutaneous or guided by echocardiography and subxiphoid pericardiostomy are effective ways of drainage in patients presenting with massive pericardial effusion and cardiac tamponade. Successful drainage has been achieved by use of percutaneous pigtail pericardial catheter [7]. Other methods of drainage include the transthoracic approach and video-assisted thoracoscopy.

We herein present our unit experience with surgical management of this condition to highlight challenges faced managing this condition with limited diagnostic facilities in the developing world. We surveyed factors that might suggest the possibility of future development of pericardial constriction.

## 2. Materials/Methods

We reviewed all cases of massive pericardial effusion and cardiac tamponade that presented to Irrua Specialist Teaching Hospital, Irrua, between February 2010 and February 2016. Irrua Specialist Teaching Hospital is a 375-bedded hospital located in a rural community and serving primarily the central, northern, and southern senatorial districts of Edo State, Nigeria.

Information was obtained from case notes, operating register, and surgeon's note. The following were documented: age, sex, clinical features suggestive of massive pericardial effusion and cardiac tamponade, investigative modalities, operative findings (thickness of pericardium, volume, and colour of fluid drained), subsequent development of recurrence, and constrictive pericarditis.

A 2D echocardiography was used to confirm the diagnosis of pericardial effusion by the presence of an echo-free space surrounding the heart. The diagnosis of cardiac tamponade was made based on the echocardiographic findings of right atrial or ventricular collapse during diastole. Chest radiograph and ECG were done in all cases.

Inclusion criteria include all patients who had moderate (10–20 mm), large (20 mm or more), and very large effusion (20 mm or more with evidence of compression of the heart).

We excluded patients with small effusion, patients with effusive-constrictive pericarditis, and two patients with cardiac tamponade who died shortly after pericardiocentesis.

Most patients had subxiphoid pericardiostomy under local anaesthesia which was augmented by conscious sedation in a handful of patients. A few patients with loculated pericardial effusion with background extensive adhesion had a limited lateral thoracostomy with creation of pericardial window.

Fluid was sent for cytology, Ziehl-Neelsen staining, and Gram staining if effluent was purulent. Pericardial biopsy was done in all cases and sent for histology.

Seventeen cases were followed up for evidence of recurrence and for development of constrictive pericarditis for a period of one to four years.

Data was entered into SPSS Version 16, statistical software package (SPSS Inc; Chicago, IL). Categorical data was calculated in frequencies and percentages. Chi-square was used for categorical variable and *p* value < 0.05 was considered statistically significant.

## 3. Result

The majority of patients (34.4%, *n* = 11) were between 31 and 40 years, followed by those between 41 and 50 years, who accounted for 18.8% of cases (Table 1). Eighteen respondents (56.2%) were males while 14 (43.8%) were females. Most of them presented with varying degrees of dyspnoea (87.5%), orthopnea (40.6%), cough (34.4%), and chest pain (28.1%). One of the patients presented with high grade fever (40–41°C) and widespread petechial haemorrhage.

Criteria for probable and definitive diagnosis of TB pericardial effusion were met in 14 patients, which accounted for the majority of cases (43.8%). This was followed by idiopathic causes, responsible for pericardial effusion in 18.7% of cases (Table 1). Out of the 32 patients studied, fourteen patients (43.8%) presented with clinical and echocardiographic features in keeping with cardiac tamponade. Electrocardiogram (ECG) showed mainly low QRS voltages.

The mean volume of fluid drained was 846 mL  ± 67 mL. The majority of respondents (53.1%) drained between 500 mL and 1,000 mL. Macroscopic appearance revealed that most of the effusions (59.4%) were haemorrhagic (Table 1). The majority of haemorrhagic effusion was probably secondary to tuberculosis (68.4%), idiopathic cause (15.8%), and uraemia (10.5%) and a case, which was suspected to be due to viral haemorrhagic fever. The patient with suspected viral haemorrhagic fever presented with high grade pyrexia (41°C) and petechial haemorrhage in addition to the haemorrhagic pericardial effusion and cardiac tamponade. Lassa polymerase chain reaction (PCR) was negative but the fever underwent resolution by lysis after initial doses of ribavirin. The patient resided in a community that is endemic for Lassa fever. Purulent pericardial effusion was observed in the 3 paediatric patients aged between 1 and 10 years. The culture grew colonies of* Staph. aureus* in 2 patients. Malignant pleural effusion was seen in 4 patients. All of the malignant effusions were secondary to metastatic carcinoma of the breast. Sixteen patients (50%) had evidence of thickened pericardium. Pericardium was considered thickened if it is ≥4 mm.

The histology revealed mainly chronic pericarditis with chronic inflammatory cells and they were either specific or nonspecific. Three out of 14 patients had histological evidence of tuberculosis. Malignant cells were seen in 1 out of all the 4 patients who presented with suspected malignant pericardial effusion.

Subxiphoid pericardiostomy was performed in 28 patients while 3 patients had limited lateral thoracotomy. Limited lateral thoracotomy was used in localized pericardial effusion, one purulent and two haemorrhagic effusions with extensive adhesions to the anterior fibrous pericardium.

There was one operative death in a patient with cardiac tamponade. Postoperative mortality from pulmonary embolism was recorded in a patient who had previous venous thromboembolism and another who died from severe renal impairment.

Seventeen cases were followed up for one to four years. No postoperative recurrence was observed. Five patients developed features of pericardial constriction. Out of these, 3 had pericardial stripping. The relationship between age at the onset of disease and nature of effusion and the development of pericardial constriction is not statistically significant; however the relationship between thickened pericardium and the development of pericardial constriction is statistically significant (*p* < 0.004) (Table 2).

## 4. Discussion

To make a definitive diagnosis of TB pericardial effusion involves demonstrating tubercle bacilli in pericardial fluid or on histologic section of the pericardium. A probable or presumed diagnosis of TB pericardial effusion involves the proof of TB elsewhere in a patient with otherwise unexplained pericarditis, a lymphocytic pericardial exudate with elevated biomarkers of TB infection, and/or appropriate response to a trial of antituberculous chemotherapy [8].

All the cases of TB pericarditis presented with infiltration of lymphocytes; however the yield of AFB on pericardial fluid and pericardial tissue was considerably low. This was obvious in the positive histology result of 3 out of 14 cases. Pericardial biopsy is positive in 10–64% of cases [9]. This was not surprising because conventional diagnostic methods used for detection of tuberculous pericarditis have been shown to be usually insensitive and require long culture periods. This may be due to the paucibacillary nature of disease and nonuniform distribution of microorganisms, coupled with the fact that precise and accurate diagnosis of pericardial effusion requires good laboratory equipment with highly trained personnel, which seems to be lacking in resource challenged settings [10]. There is therefore a need to incorporate other tests that are very sensitive for detecting TB pericardial effusion.

Tuberculosis is the most likely common cause of massive pleural effusion and cardiac tamponade and it is responsible for 43.8% of cases. Our finding is in keeping with findings of Agner and Gallis, who observed that tuberculosis and malignant effusion were more likely to cause large pericardial effusion, effusion causing haemodynamic compromise compared to those secondary to idiopathic pericarditis [11]. Other studies done in regions with high endemicity for TB also revealed TB as the most common cause of pericardial effusion [12, 13]. All our patients suspected to have TB pericardial effusion received an initial 4-drug therapy for 2 months (isoniazid, rifampicin, pyrazinamide, and ethambutol) followed by isoniazid and rifampicin for the remaining 4 months. The findings of TB as the most common cause of pericardial effusion contrasts the findings in developed countries. In a study by Colombo et al., the most frequent causes of pericardial effusion were neoplastic (36%), idiopathic (32%), and uraemic (20%) whereas in the series of 57 patients investigated by Corey et al. the most common diagnoses were malignancy (23%), viral infection (14%), radiation induced inflammation (14%), collagen-vascular disease (12%), and uraemia (12%) [3, 14]. Sagristà-Sauleda et al. documented acute idiopathic pericarditis as the most common cause of massive pericardial effusion and this accounted for 20% of cases. This was followed by iatrogenic effusion (16%), neoplastic effusion (13%), and chronic idiopathic pericardial effusion (9%) [15]. A more recent study by Abdallah and Atar revealed that the most frequent aetiology of large symptomatic pericardial effusion was idiopathic [36% (77% with a clinical diagnosis of pericarditis)], followed by malignancy (31.4%), ischemic heart disease (16.3%), renal failure (4.6%), trauma (4.6%), and autoimmune disease (4.6%) [16].

In our study, TB pericardial effusion was the most likely cause of haemorrhagic pericardial effusion. In a study, haemorrhagic pericardial effusion was secondary to TB in 80% of patients [17]. Haemorrhagic pericardial effusion has been associated with neoplasia and poor survival in some studies, whereas others have implicated iatrogenic disease, malignancy, atherosclerotic heart disease, and idiopathic diseases as the major causes of haemorrhagic pericardial effusion [14, 18].

Purulent pericardial effusion was observed only in 3 paediatric patients. 1.2 L of pus was drained from the pericardium of one of these patients. Purulent pericarditis is a suppurative complication of bacterial infection of the pericardial space that can arise as a result of direct extension from an adjacent infection. This is supported by one of the patients who presented with extensive pyomyositis.* Staphylococcus aureus* is the most commonly identified pathogen as in our study, though other organisms as nontypeable* H. influenzae* (NTHi) and* Streptococcus pneumoniae* have been isolated [19–21].

The most common symptom was dyspnoea. Orthopnea was particularly observed in patients who presented with cardiac tamponade. Other studies have reported dyspnoea as the most common symptoms [11, 12]. Many patients (43.8%) presented with history and echocardiographic features in keeping with cardiac tamponade because generally in the developing world most of our patients present late.

The majority of pericardial effusion was drained by subxiphoid pericardiostomy; hence we strongly advocate this technique. Other researchers have also advocated this route [22–24]. We performed 28 out of 32 cases under local anaesthesia. Palatianos et al. used general anaesthesia in 35 out of 42 cases that they performed on [22]. Subxiphoid pericardiostomy offers rapid access to the pericardium and has low morbidity and excellent long term results as noted in other studies [23]. It is also performed under local anaesthesia and contamination of pleural space especially in cases of purulent pericarditis is avoided. It is also easy to obtain satisfactory pleural biopsy.

We used limited lateral thoracostomy on few occasions, when there is extensive adhesion anteriorly. This is to avoid inadvertent entry to the heart. Thoracotomy has been shown to result in a higher incidence of respiratory complications, as defined by the presence of pneumonia, pleural effusion, prolonged ventilation, and need for reintubation. Thoracotomy also has a longer mean hospital stay [25]. We have never considered video-assisted thoracoscopy because we do not have the facilities. We did not perform initial pericardiocentesis on most cases of cardiac tamponade because we have no delay operating on them. Most of our effusions are haemorrhagic and it may be difficult to differentiate a haemorrhagic pericardial effusion ab initio and one secondary to myocardial puncture especially when the procedure is performed without echocardiographic guidance. Pericardiocentesis is also not without risk of atrial and ventricular arrhythmias, vasovagal episodes, and pneumothorax.

We did not record any case of recurrence following drainage. This contrasts the findings of Sarigül et al., who recorded recurrence of 10.2%, and recurrence was most commonly observed in uraemic patients [13]. Our zero recurrence may be related to our protocol for management of these patients, as we ensure drainage less than 50 mL before removal of tubes. The normal pericardial sac contains 10–50 mL of pericardial fluid, which acts as a lubricant between the pericardial layers. We also ensured that patient adhered strictly to the management of the primary pathology. Shahbaz Sarwar and Fatimi recorded recurrence in 32 out of 99 patients treated for pericardial effusion. TB was the most common cause of recurrent effusion in their study [26]. In a study by Mueller et al., 18% had recurrent pericardial effusion [24].

A constrictive physiology can develop within months and years after pericardiostomy. Five patients developed constrictive pericarditis. Out of this, 3 had pericardiectomy. They were more likely to present with echocardiographic findings of thickened pericardium. Studies have also that a high risk of constriction has been observed in cases of purulent pericarditis, tubercular pericarditis, and radiation pericarditis [21, 27, 28]. Late pericardial constriction has also been noted in a patient with idiopathic pericardial effusion [24]. We followed up our cases of purulent pericarditis and we did not encounter features of constriction.

Our study is limited by the number of cases seen over the 6-year period. A result from a sizeable study population may be more relevant.

## 5. Conclusion

Massive pericardial effusion and cardiac tamponade are largely secondary to tuberculosis in the developing world. Subxiphoid pericardiostomy is a satisfactory method of drainage of pericardial effusion and postoperatively those with thickened pericardium need to be closely monitored.

## Figures and Tables

**Table 1:** Demographic characteristics of respondents.

Demographic variables	Number	%
*Age*		
0–10	3	9.4
11–20	2	6.2
21–30	5	15.6
31–40	11	34.4
41–50	6	18.8
51–60	3	9.4
>60	2	6.2

*Sex*		
Male	18	56.2
Female	14	43.8

*Causes *		
Bacterial infection	3	9.4
Idiopathic	6	18.7
Malignant	4	12.5
Tuberculosis	14	43.8
Steroid-resistant nephritic syndrome	1	3.1
Suspected haemorrhagic fever	1	3.1
Uraemia	3	9.4

*Volume of fluid drained intraoperatively*		
<500	5	15.6
500–1000	17	53.1
>1000	10	31.3

*Nature of fluid*		
Serous	10	31.3
Haemorrhagic	19	59.4
Purulent	3	9.3

**Table 2:** Effect of age, pericardial thickness, and nature of effusion on development of pericardial constriction.

Variables		Development of constrictive pericarditis (CP)		*p* value
		Developed CP	Has not developed CP	
*Age*				0.409
0–10		—	3	
11–20		—	2	
21–30		1	3	
31–40		1	9	
41–50		2	2	
51–60		1	1	
>60		—	2	

*Thickened pericardium *	*No*			0.004
Present	12	5	7	
Absent	15	0	15	

*Nature of effusion *	*No*			0.296
Haemorrhagic	16	4	12	
Nonhaemorrhagic	11	1	10	
